# The Role of Income and Food Prices in Diet-Related Greenhouse Gas Emissions in China: A Path towards a Sustainable Diet

**DOI:** 10.3390/foods11244051

**Published:** 2022-12-14

**Authors:** Jiao Chen, Funing Zhong

**Affiliations:** College of Economics and Management, Nanjing Agricultural University, Nanjing 210095, China

**Keywords:** food demand, income, food prices, greenhouse gas emissions, sustainable diet

## Abstract

China has undergone rapid economic growth and diet transition over the past decades, along with environmental challenges. To help to achieve carbon peaking by 2030, this study investigates the time-variable diet-related greenhouse gas emissions (GHGEs) from 2000 to 2020 and examines the responses of food demands and diet-related GHGEs to an increase in per capita income and animal-based food prices. Based on the Almost Ideal Demand System model, addressing the problem of the endogeneity of food expenditure, we estimate the demand elasticities for six food groups and analyze their changing trends with time. Then, we perform two projections. One is to project the impacts of income growth on food demands and diet-related GHGEs, taking into consideration dynamic income elasticities as per capita income increases; the other is to project the effects of an increase in prices of different animal-based foods on food demands and diet-related GHGEs. Our results reveal that diet-related agricultural GHGEs show a continued increase in the short term, but the increased effect tends to decline gradually. The dominant driver of increased diet-related GHGEs is the increased consumption of beef, mutton, and pork. However, an increase in prices of beef and mutton; pork; and poultry and eggs can reduce diet-related GHGEs, while a decrease in the prices of aquatic products can also deliver a significant decrease in GHGEs. The results imply that policymakers should take an interest in the environmental impacts of diet transition and promote a more sustainable diet structure.

## 1. Introduction

The agricultural sector is an important contributor to anthropogenic greenhouse gas emissions (GHGEs) [1]. GHGEs from on-farm production and related land use changes account for about 20% to 25% of the total emissions from all human activities [2]. This contribution is even more striking for non-CO_2_ GHGEs. For example, crop and livestock production within the farm gate contribute more than 50% of the total anthropogenic methane (CH_4_) and 75% of the nitrous oxide (N_2_O) emissions [3,4]. Besides, emissions from pre-production, such as fertilizer and pesticide manufacturing, also increase these emissions. Hence, mitigating agricultural GHGEs has attracted wide public concern.

In addition to the mitigation strategies in food supply chains, dietary patterns are also critical to mitigating agricultural GHGEs through their impact on supply activities. Many studies have explored a series of approaches to reduce agricultural GHGEs from the supply side, such as technological innovation and production management [1]. From another perspective, the food demand determines the food production, in regards to both quantities and structures, which is related to the agricultural GHGEs [5,6]. With economic development and population growth, the food demand is increasing, and the structures tend towards animal-based food [7]. Especially in developing countries, the rapid per capita income growth has noticeably improved diets [8]. Thus, it is interesting to study the changes in residents’ food demands and diet-related agricultural GHGEs over the past decades and project their responses to income growth and the increased prices of animal-based foods.

China is an interesting and important case in regards to this problem. First, China is a traditional agricultural country, which feeds about 20% of the global population. The agricultural GHGEs account for 17% (according to the Initial National Climate Change Bulletin of the People’s Republic of China) of the total anthropologic GHGEs in China, and the ratio is higher than the average global level [3]. This study will help us explore how to reduce agricultural GHGEs from the demand side. Second, China is a populous country with enormous economic development; thus, the food demand has experienced significant changes in quantity and structure [9,10]. From 1987 to 2017, the diet-related GHGEs increased by 64%, driven by a more meat-intensive diet [11]. Thus, estimating the food demand characteristics and projecting the changing trends is important for both food security and a sustainable environment. Last, to show responsibility for sustainable development, the Chinese government aims to achieve carbon peaking by 2030 and carbon neutrality by 2060. The result of this study will contribute to achieving the goals from the perspective of the food demand side.

A great deal of work has been done to evaluate diet-related GHGEs and propose strategies to reduce GHGEs. Previous studies have calculated the GHGEs from plant- and animal-based food [12] and evaluated diet-related GHGEs at national [11,13,14], provincial [15], and household levels [9,16,17]. Further, the driving factors, such as Engel’s coefficient, urban/rural status, and demographic factors of increased diet-related GHGEs, have been analyzed [13,14,17]. Some studies also compared the current Chinese diet with Western diets or diets recommended by the Chinese Dietary Guidelines, simulating the future changes in diet-related GHGEs under different diet scenarios [9,18,19]. These results showed that a healthier diet with less meat consumption can facilitate a reduction in agricultural GHGEs [9,18]. In this regard, some Western scholars suggested levying a carbon tax on animal-based foods, which can efficiently reduce diet-related GHGEs [20,21,22]. However, few studies evaluated the impacts of income growth or a rise in food prices on diet-related GHGEs in China based on demand models. Based on previous studies, this study investigates the time-variable GHGEs from 2000 to 2020 and further examines the responses of food demands and diet-related GHGEs to an increase in per capita income and prices of various animal-based foods.

Many studies estimated food demand elasticities using demand models or meta-analysis, some of which further projected the food demands in the future. These results showed that income responsiveness was higher for meat consumption, and meat demand would continue to increase with income growth [23,24,25]. However, the income elasticities of most foods tend to decline with income increases, while the own-price elasticities for some foods tend to be more elastic [25,26]. This means that bias between food demand projections based on dynamic income elasticities and results based on constant elasticities are substantial, and the bias will gradually increase over time [26]. Hence, this study estimates the time-variable demand elasticities and performs the projections considering the dynamic demand elasticities as per capita income increases.

This study aims to estimate the dynamic changes of food demand elasticities with time-series data, and project the responses of food demands and diet-related agricultural GHGEs to an increase in per capita income and animal-based food prices. This study fills the gap in the literature by (1) projecting the impacts of a rise in income and animal-based food prices on diet-related GHGEs considering the change in demand elasticities; (2) analyzing the change trends in demand elasticities with time-series data. We employed the data from the China Statistical Yearbook (2001~2021) and divided the residents’ consumed foods into six groups. First, we clarified the food demand characteristics and related agricultural GHGEs from 2000 to 2020. Then, we used the Almost Ideal Demand System (AIDS) model to estimate the demand elasticities of six food groups and analyzed their dynamic changes. Last, we performed two projections. One is to simulate the impacts of income growth on food consumption and diet-related GHGEs considering the dynamic changes of income elasticities; the other is to project the responses of food consumption and diet-related GHGEs to an increase in prices of different animal-based foods.

The remainder of the paper is organized as follows. Section 2 introduces the methodologies and data, including the conceptual framework, demand model, and calculations of diet-related agricultural GHGEs. Section 3 presents the results. Section 4 and Section 5 discuss and conclude the study, respectively.

## 2. Materials and Methods

### 2.1. Conceptual Framework

**Figure 1:**
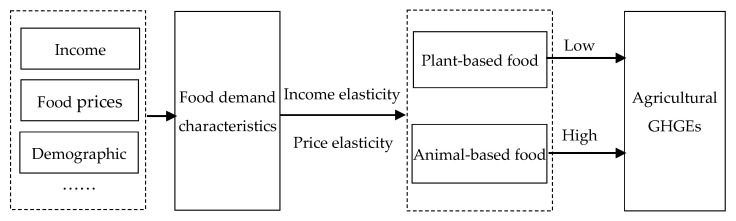
Conceptual framework of changing food demands and related GHGEs.

The conceptual framework of this study is shown in Figure 1. Per capita disposable income and food prices are the main factors impacting residents’ food demands. Income elasticity of demand is an economic measure of how responsive the quantity demanded for a good or service is to a change in income under the condition that prices and other factors remain unchanged. For a normal good, the demanded quantity is increased as income increases at each price level; that is, the elasticity value is positive. However, for an inferior good, income growth reduces the demand at each price level. The growing income improves people’s living standards and changes their desires and preferences for foods; thus, the demand elasticities for various food groups are under dynamic changes [25,26]. Price elasticity is an economic measure of how responsive the quantity demand for a good or service is to a change in the price of a good or service, when other factors remain constant. Relative changes in the prices of one food can cause compensated effects or substituted effects for other foods. In addition to per capita income and prices, the population structures also impact food demands, as people of various ages have different dietary habits and caloric requirements. In a word, with the development of economy and population structures, both food demands and demand elasticities exhibit dynamic change.

For different agricultural products, the differences in greenhouse gases released in agricultural production processes are substantial. Generally, the carbon emission intensity per unit mass of animal-based food is much higher than that of plant-based food, especially for ruminant food such as beef and mutton [27]. This means that diet-related agricultural GHGEs might increase if the residents’ diet changes from plant-based food to animal-based food. If GHGEs from increased animal-based food are less than those from reduced plant-based food, then total diet-related agricultural GHGEs might be reduced.

### 2.2. Demand Model

To estimate the demand elasticities for various food groups, we employed the Almost Ideal Demand System (AIDS) model derived by Deaton and Muellbauer [28]. The budget share form of AIDS is defined as follows:

(1) $$w_{i}=\alpha_{i}+\sum_{j}\gamma_{ij}\ln p_{j}+\beta_{i}\ln\left(\frac{m}{P}\right)+\mu_{i}$$

where P is a price index P=a(p), $\log a(p)=\alpha_{0}+\sum_{k}^{}\alpha_{k}\log p_{k}+\frac{1}{2}\sum_{k}^{}\sum_{j}^{}\gamma_{kj}^{*}\log p_{k}\log p_{j}$. i, and j refers to the food group. $w_{i}$ is the budget share of the ith food group, which is $w_{i}=\frac{p_{i}q_{i}}{m}$. $p_{j}$, and $q_{i}$ refers to the food price and quantity, respectively. m represents the total food expenditure. $\alpha_{i}$, $\beta_{i}$, and $\gamma_{ij}$ are parameters to be estimated. $\mu_{i}$ is the error term. The adding-up, homogeneity, and Slutsky symmetry restrictions on the parameters are as follows:

(2) $$\sum_{i}\alpha_{i}=1,\;\sum_{i}\beta_{i}=0,\;\sum_{i}\gamma_{ij}=0,\;\gamma_{ij}=\gamma_{ji}$$

In addition to price and income, demographic structures also affect food demand [29]. There are many ways to include demographic variables in the demand model. Here, according to Lecocq and Robin [30], the demographic structure variable enters the demand system through α. The total food expenditure is the sum of expenditures regarding an individual food group, and the individual food expenditure is the result of multiplying food prices by consumed quantities. Hence, these expenditures might be jointly endogenous. To address the problem of endogeneities in the total expenditure, we followed Blundell and Robin [31] by using instrumental-variable and augmented regression. There are two steps in this method. First, we estimate a model for the total expenditure using income as an instrumental variable (lny). Second, we incorporate the residuals as an extra control variable in Equation (1). The AIDS was estimated by the iterated linear least-squares (ILLS) estimator [31], and the command comes from Lecocq and Robin [30].

The food expenditure and price elasticities can be derived by the following equations:

(3) $$e_{i}=1+\frac{\beta_{i}}{w_{i}}$$

(4) $$e_{ij}^{u}=-\delta_{ij}+\frac{1}{w_{i}}\left(\gamma_{ij}-\beta_{i}\left(\alpha_{j}+\sum_{l}^{}\gamma_{jl}\ln p_{l}\right)\right)$$

where $\delta_{ij}$ is the Kronecker delta. $\delta_{ij}=1$ when i=j, or $\delta_{ij}=0$ when i≠j.

Further, to measure the income elasticities of various food groups, we used Working’s model [32] to analyze how consumers allocate shares between foods and non-foods. The total household food expenditure share function is set as follows:

(5) $$W_{F}=\beta_{0}+\beta_{1}\ln y+\mu$$

where W represents the share of total food expenditure in the per capita disposable income, y refers to per capita disposable income, and μ is the error term.

Then, the income elasticity of the total foods can be expressed as follows:

(6) $$\eta =\beta_{1}/W_{F}+1$$

The unconditional (income) elasticity for various foods can be derived by:

(7) $$\eta_{i}=\eta e_{i}$$

### 2.3. Calculation of Diet-Related Agricultural GHGEs

This study mainly focuses on the direct and indirect GHGEs in the agricultural production process; the GHGEs released from storing, transporting, and cooking are not included. The carbon emission intensities of major food groups and their references can be found in Table 1. The data and research methods used in these two publications can better reflect the carbon emission intensity of agricultural products produced in mainland China. Lin et al. calculated the carbon emission intensities of China’s agricultural products using a hybrid economic input-output and life cycle assessment (EIO-LCA) model, and their data came from the National Cost-Benefit Compilation of Agricultural Products, the China Rural Statistical Yearbook, and the China Greenhouse Gas Inventory [27]. Liu and Che calculated the carbon intensities of China’s aquatic products using the data from the China Fishery Statistical Yearbook, with the methods introduced by the Oak Ridge National Laboratory [33].

According to the agricultural carbon emission intensity coefficients and per capita annual consumption of various food groups, the per capita agricultural GHGEs related to individual food and total food consumption over the years can be expressed as follows, respectively:

(8) $$GHG_{ki}=car_{i}q_{ki}$$

(9) $$GHG_{k}=\sum_{i=1}^{n}car_{i}q_{ki}$$

where k represents the year, $car_{i}$ is the agricultural carbon emission intensity of each food item (Table 1), and $q_{ki}$ represents the per capita consumed quantities of food i in a year k.

### 2.4. Data

The data for per capita food consumption, disposable income, demographic structure, and the consumer price index come from the China Statistical Yearbook (2001–2021), and the food market prices from 2003 to 2020 come from the China Yearbook of Agricultural Price Survey (2004–2021). We used the proportion of residents aged 15 to 64 years as the demographic structure variable. Due to missing data, the food market prices before 2003 are derived by the consumer price sub-index and food market prices in 2003. Both per capita income and food expenditure have flattened the consumer price index based on the data from the year 2000. As the statistical criterion of urban residents’ grain consumption before 2012 and after 2013 is processed grain and raw grain, respectively, we followed the methods of Cao et al. [34] and used the conversion coefficient (from 2013 to 2016, the per capita raw grain consumption of urban residents was reduced from 121.3 kg to 111.9 kg, so the average annual reduction was 3.13 kg. Cao et al. [34] used the average trend method to evaluate the consumed quantity in 2012. The evaluated result was 124.4 kg raw grain, while the original data was 78.8 kg processed grain. Hence, Cao et al. [34] determined that the conversion coefficient between processed grain and raw grain was 0.63) between processed grain and raw grain (0.63). As animal-based food is generally more carbon-intensive than plant-based food, and various animal-based foods show a substantial difference in agricultural carbon emission intensities (Table 1), we divided the animal-based foods according to their carbon intensity. Finally, the foods were divided into six groups, including staple foods; vegetables and fruits; pork; beef and mutton; poultry and eggs; and aquatic products.

## 3. Results

### 3.1. Descriptive Analysis

Since the 21st century, China has experienced rapid growth in per capita disposable income and food expenditure (Table 2). The per capita income in 2020 was more than 5.48 times as much as it was in 2000. Per capita income grew at an annual rate of 10.56% between 2000–2010 and 7.23% between 2010–2020, respectively. However, the total food expenditure grew at a solid annual rate of 5.02% and 4.97% between 2000–2010 and 2010–2020, respectively. Thus, the share of total food expenditure in per capita disposable income decreased at an annual rate of 5.01% and 2.20% between 2000–2010 and 2010–2020, respectively. This shows that the change rates of both per capita disposable income and the shares of food expenditure are decreasing. At the same time, the rapid income growth has dramatically improved Chinese diets. Note that the shares of animal-based food expenditure in the total food expenditure grew at an annual growth rate of 2.32% between 2010–2020, faster than that between 2000–2010. This indicates that the growth rate of the animal-based food expenditure share is still rising with an increase in income, and the share has been over 50% since 2019.

**Figure 2:**
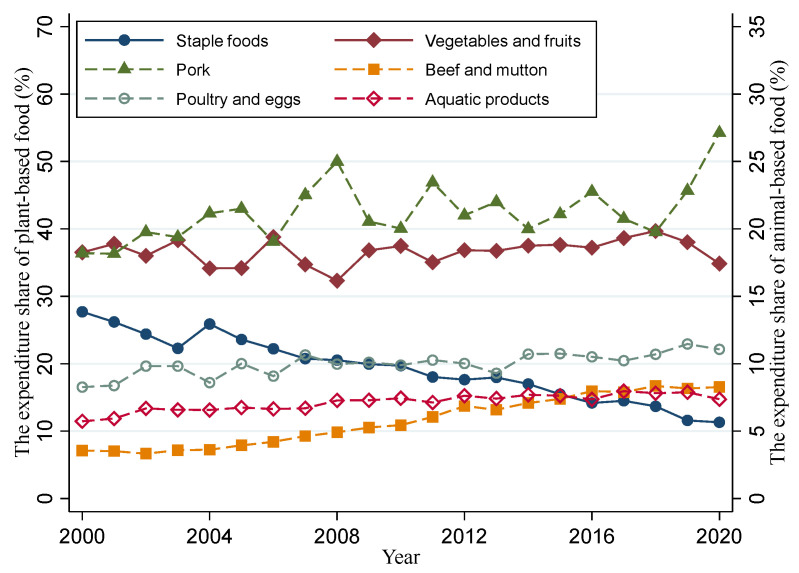
Per capita expenditure shares of various food groups from 2000 to 2020.

Further, we analyzed the expenditure shares of each food group from 2000 to 2020 in China (Figure 2). Among plant-based foods, vegetables and fruits retain a stable expenditure share (30–40%), while the expenditure share of staple foods shows a downward trend, from 27.7% in 2000 to 11.3% in 2020. Among animal-based foods, pork has the highest expenditure share and shows a fluctuating upward trend. Especially, as the outbreak of African swine fever increased pork prices, the pork expenditure share increased sharply, even though the consumption of pork had been decreasing since 2019 (Table 3). The expenditure share of poultry and eggs and aquatic products shows a slow upward trend. However, although the expenditure share of beef and mutton was the lowest at the beginning of the 21st century, it showed a remarkable speed of growth over the past two decades.

**Figure 3:**
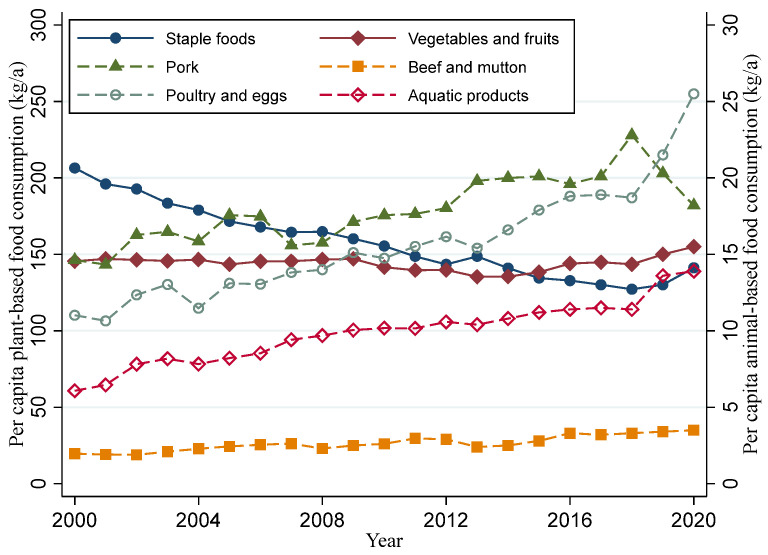
Per capita food consumption of various food groups from 2000 to 2020.

**Figure 4:**
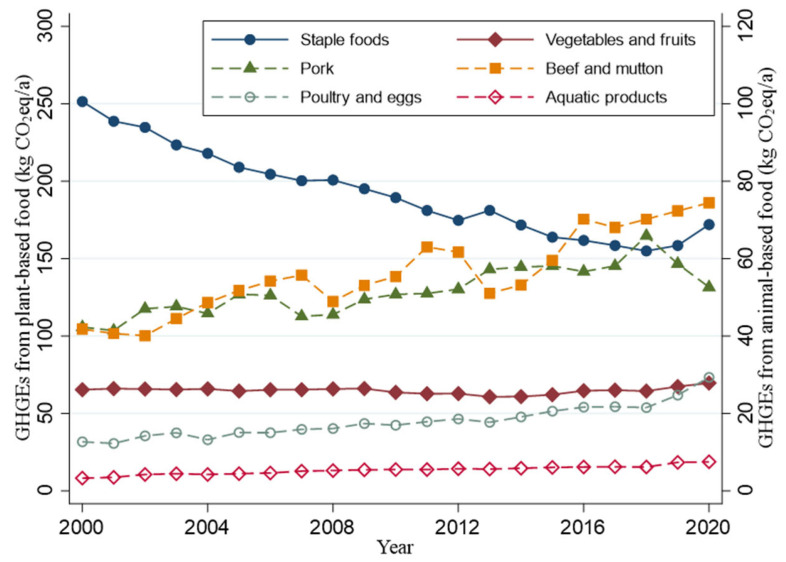
Per capita diet-related agricultural GHGEs from 2000 to 2020.

Figure 3 and Figure 4 show per capita food consumption and their related agricultural GHGEs from 2000 to 2020, respectively. The consumed quantities of plant-based foods and their corresponding agricultural GHGEs show a downward trend, while all animal-based food consumption and corresponding agricultural GHGEs exhibit an upward trend. Especially for poultry and eggs and aquatic products, the consumed quantities in 2020 were more than two times as much as those in 2000. Although the consumption of beef and mutton is the lowest, the corresponding agricultural GHGEs are relatively high. Since 2015, the GHGEs from beef and mutton consumption had surpassed those from pork, ranking first among animal-based foods.

### 3.2. Demand Elasticities

To conserve space, only expenditure, income, and price elasticities every four years are shown in Table 3 and Table 4, respectively. Expenditure and income elasticities for all foods are positive and statistically significant at 0.01%, except for staple foods. This indicates that staple foods are inferior goods, while others are normal goods for Chinese residents. Moreover, all expenditure and income elasticities show a downward trend with time, which is associated with the continued increased disposable income per capita. This means that a 1% increase in income will lead to a greater decrease in the demand for staple foods and a lower increase in the demand for other foods, as income increases in China in future. Last, among all foods, the income elasticities for beef and mutton are the highest, followed by those for aquatic products. This implies that income growth is inclined to drive a greater increase in the consumption of beef, mutton, and aquatic products. However, a larger increase in beef and mutton means a rapid increase in diet-related agricultural GHGEs.

The own-price elasticities for vegetables, fruits, pork, beef, mutton, and aquatic products are negative and statistically significant, while those for staple foods, poultry and eggs are not sensitive to price changes (Table 4). Different from expenditure and income elasticities, there is no evident trend in price elasticities, and the differences across years are small. Compared to the average price elasticities between 2000–2010 and 2010–2020, we found the absolute value of price elasticities of vegetables, fruits, pork, beef, mutton, poultry, and eggs increased, while those of aquatic products decreased. This indicates that animal-based foods, except for aquatic products, are more sensitive to price changes with time, and perhaps we can use prices to manage the animal-based food demand. Among all animal-based foods, aquatic product consumption is the most sensitive to price changes, followed by that of beef and mutton.

### 3.3. Projections

In this section, we perform two projections. One is to project the response of food demands and corresponding agricultural GHGEs to a 1% increase in per capita income. This is based on income elasticities for individual food groups (Table 5). The other is to project the change in food demands and their associated agricultural GHGEs resulting from a 1% increase in the prices of animal-based foods. This is based on uncompensated prices for individual food groups (Appendix A).

Before performing the first projection, we assume that the food prices are to be constant. According to the above results, the income elasticities show a downward trend as income increases; hence, here we perform three scenarios. S1 shows the average income elasticities from 2000 to 2020; S2 includes the average income elasticities from 2010 to 2020; S3 reduces the average income elasticities from 2010 to 2020 by 10%, according to the changing trend in income elasticities (Table 5). Before performing the second projection, we assume that the consumer preferences and market development are constant. As various animal-based foods have different carbon emission intensities, here we include four scenarios. SS1, SS2, SS3, and SS4 represent a 1% increase in the prices of pork; beef and mutton; poultry and eggs; and aquatic products, respectively.

Table 6 shows the projected results of the responses of food demand and related GHGEs to a 1% increase in per capita income. The final change in food consumption is related to the originally consumed quantities and income elasticities. Hence, the changes in per capita plant-based food consumption are larger than those for animal-based food consumption. As the income elasticity of staple foods is negative, the income growth reduced its consumption and the related GHGEs. Among the animal-based food, the greatest increase in GHGEs was for beef and mutton consumption, followed by pork consumption under three scenarios. The per capita total increased agricultural GHGEs are 1.135 and 0.236 kg CO_2_eq under S1 and S2, respectively, while these decrease by 0.076 kg CO_2_eq under S3. This implies that the decreased income elasticities drive a lower increase in total diet-related agricultural GHGEs, and even reduce them.

Table 7 displays the projected results of the responses of food demand and related GHGEs to a 1% increase in the prices of animal-based foods. As there are substituted and compensated effects across various food groups, the impacts of a 1% increase in different animal-based foods on total diet-related agricultural GHGEs are diverse. The largest decrease in total diet-related agricultural GHGEs is noted for SS2, followed by SS1 and SS3. However, if we increase the prices of aquatic products, the total diet-related GHGEs increase by 1.300 kg CO_2_eq. This is due to the substitute effects of staple foods, beef, and mutton. This result indicates that we might reduce GHGEs by raising the price of beef and mutton and cutting the price of aquatic products.

## 4. Discussion

Given the continued economic growth and diet transition, this study analyzed the time-variable demand elasticities and explored the responses of food demand and diet-related agricultural GHGEs to changes in per capita income and food prices. Consistent with the results of previous studies [9,35], we found that the share of animal-based food expenditure in the total food expenditure, quantities of animal-based food consumption, and diet-related GHGEs from animal-based foods showed an upward trend during the past two decades. For this, per capita income growth is the main driving factor, and demographic transition and urbanization also play a role [13,29,36]. Although the consumption of beef and mutton is not much, the corresponding GHGEs are high and have become the highest among those from animal-based foods since 2015. The high carbon emission intensity of ruminant meat and the growing appetite for beef and mutton can explain this effect [27,37].

Our results show that income elasticities for most foods tend to decline, which are consistent with previous findings, although the exact elasticity values from various studies are different due to various data sources, food categories, and methods applied (Appendix B). The changing trend of income elasticities agrees with our expectations. Moreover, our results reveal that own-price elasticities for vegetables and fruits; pork; beef and mutton; and poultry and eggs are more elastic as per capita income increases. However, Liu and Zhong [38] showed that own-price elasticities for rice and flour became more sensitive to price changes over time. This may be because Liu and Zhong [38] used samples from 1986 to 2002, during which time people were still searching to obtain enough calories instead of looking for diverse foods. One explanation for these increased price-elastic elasticities may be that the economic and market development provide consumers with more choices, more substitution possibilities, and price-elastic demand [25]. Animal-based foods, especially beef and mutton, are more sensitive to income growth than plant-based foods. This result agrees with previous findings, which imply that animal-based food consumption will grow faster than plant-based food consumption as income increases [39,40]. Among all animal-based foods, aquatic product consumption is the most price-elastic, followed by beef and mutton consumption, and pork consumption. This indicates that policymakers might reduce GHGEs through food demand management by food price adjustment, while assuring residents’ nutrition intake.

Further, our projections show that the demand for all foods, except staple foods, will increase as per capita income increases. This is according to the finding of Zheng et al. [41], who revealed that food security in China has been transformed into feed grain security. Further, the increased food demands decline with income growth due to the decreased income elasticities. Hence, the increased diet-related agricultural GHGEs also tend to decline, and may even be negative. Besides, if we increase the prices of beef and mutton, pork, and poultry and eggs and reduce the prices of aquatic products, the diet-related agricultural GHGEs can be noticeably reduced. This is according to Western research, which showed that imposing a tax on several animal-based foods can effectively mitigate GHGE [22,42]. However, as China is still a developing country, any policy aiming to reduce GHGEs should guarantee food security and residents’ nutrition intake. Hence, China may combine a tax and subsidy policy to promote a sustainable diet.

From the demand side, a single measure is also not enough to achieve carbon neutrality and mitigate the projected increase in environmental burden. In addition to improving diet, international trade and reducing food waste and food processing are also expected to deliver emission reductions [5,6,43]. For example, Kong et al. found that if we decrease food waste and food processing by 10%, the total agricultural GHGEs can be reduced by 1.59% [44].

There are several limitations in this study, which should be further addressed in the future. First, the heterogeneities of regional disparity and the urban-rural gap should be further analyzed. Second, the data from the China Statistical Yearbook only include residents’ food consumption at home, ignoring the food consumption away from home. As meat demand, especially beef consumption, away from home grows more than proportionally to total food consumption [45], our result might underestimate diet-related GHGEs and their responses to an increase in income and animal-based food prices.

## 5. Conclusions

This study investigated diet-related agricultural GHGEs in China and their responses to an increase in per capita income and animal-based food prices. The AIDS model, considering the issue of food expenditure endogeneity, was used to estimate demand elasticities for six food groups from 2000 to 2020. Then, the impacts of an increase in per capita income on food demands and diet-related agricultural GHGEs are projected considering the trend in the changes in income elasticities. Meanwhile, we also projected the responses of diet-related agricultural GHGEs to an increase in the prices of pork, beef and mutton, poultry and eggs, and aquatic products, respectively.

Our results indicate that the food demand and diet-related agricultural GHGEs will increase in the short term, but the increased effect tends to decline gradually. In the long term, the total diet-related agricultural GHGEs might decrease due to a lower increase in animal-based food and a greater decrease in staple foods. The dominant driver of increased diet-related GHGEs is the increased consumption of beef and mutton and pork. However, increases in prices of beef and mutton, pork, and poultry and eggs can reduce diet-related agricultural GHGEs, while an increase in the price of aquatic products has the opposite effect.

Along with remarkable economic development, China is also facing environmental challenges. On the road to achieving carbon peaking and carbon neutrality, this study has important implications for policymakers. With the continued increase in per capita income, although diet transition improves residents’ nutritional status, the government should pay attention to the environmental impacts driven by the rapid increase in animal-based food demand. In addition to the mitigation strategies from the supply side, the government can reduce GHGEs through food demand management from the demand side, while ensuring residents’ nutritional intake, for example, by subsidizing low-carbon food or taxing carbon-intensive food to promote a more sustainable diet structure.

## Figures and Tables

**Table 1:** Agriculture carbon emission intensities of major food categories.

Food Groups	Carbon Intensity (kg CO_2_eq/kg)	References
Staple foods	1.218	[27]
Vegetables and fruits	0.449	[27]
Pork	2.890	[27]
Beef and mutton	21.265	[27]
Poultry and eggs	1.150	[27]
Aquatic products	0.540	[33]

**Table 2:** Per capita income and food expenditure in China between 2000–2020.

Year	y (CNY)	m (CNY)	W_F_ (%)	W_MF_ (%)	De *(*%)
2000	3711.8	823.7	22.19	35.76	70.1
2001	4030.7	815.6	20.23	36.01	70.4
2002	4524.1	853.8	18.87	39.61	70.3
2003	4939.7	899.2	18.20	39.38	70.4
2004	5374.6	996.4	18.54	39.96	70.9
2005	5954.9	1023.0	17.18	42.22	72.0
2006	6644.2	1039.9	15.65	38.98	72.3
2007	7532.3	1152.0	15.29	44.52	72.5
2008	8252.4	1231.4	14.92	47.16	72.7
2009	9168.7	1293.0	14.10	43.23	73.0
2010	10,125.5	1343.7	13.27	42.81	74.5
2011	11,264.8	1526.2	13.55	46.91	74.4
2012	12,545.2	1566.5	12.49	45.52	74.1
2013	13,360.6	1585.9	11.87	45.30	73.9
2014	14,426.4	1592.4	11.04	45.49	73.4
2015	15,496.3	1648.3	10.64	46.87	73.0
2016	16,475.4	1707.7	10.37	48.60	72.5
2017	17,682.6	1653.8	9.35	46.85	71.8
2018	18,821.6	1684.8	8.95	46.63	71.2
2019	19,913.5	1950.1	9.79	50.36	70.6
2020	20,349.5	2183.1	10.73	53.83	68.6

Note: y and m represent per capita disposable income and total food expenditure, respectively. W_F_ represents the share of total food expenditure in disposable income.W_MF_ represents the share of animal-based food expenditure in the total food expenditure. De refers to the proportion of residents aged from 15 to 64 years.

**Table 3:** Expenditure and income elasticities for various food groups from 2000 to 2020.

Year	2000	2004	2008	2012	2016	2020	2000–2010	2010–2020
Income elasticities for total food consumption								
	0.694 ***	0.633 ***	0.544 ***	0.455 ***	0.344 ***	0.366 ***	0.603 ***	0.387 ***
	(0.013)	(0.015)	(0.019)	(0.022)	(0.027)	(0.026)	(0.016)	(0.025)
Expenditure elasticities for various foods								
F1	−0.761 ***	−0.877 ***	−1.220 ***	−1.618 ***	−2.406 ***	−3.074 ***	−1.049 ***	−2.170 ***
	(0.213)	(0.230)	(0.255)	(0.321)	(0.446)	(0.524)	(0.244)	(0.398)
F2	1.113 ***	1.123 ***	1.129 ***	1.114 ***	1.111 ***	1.118 ***	1.115 ***	1.111 ***
	(0.213)	(0.230)	(0.242)	(0.214)	(0.208)	(0.223)	(0.216)	(0.209)
F3	1.763 ***	1.665 ***	1.580 ***	1.663 ***	1.628 ***	1.532 ***	1.695 ***	1.647 ***
	(0.400)	(0.350)	(0.299)	(0.349)	(0.323)	(0.281)	(0.363)	(0.336)
F4	4.776 ***	4.439 ***	3.684 ***	2.981 ***	2.732 ***	2.591 ***	4.145 ***	2.769 ***
	(1.151)	(1.026)	(0.815)	(0.602)	(0.499)	(0.492)	(0.958)	(0.521)
F5	1.815 ***	1.747 ***	1.680 ***	1.685 ***	1.639 ***	1.622 ***	1.712 ***	1.641 ***
	(0.482)	(0.442)	(0.396)	(0.407)	(0.370)	(0.373)	(0.420)	(0.375)
F6	2.444 ***	2.334 ***	2.240 ***	2.186 ***	2.129 ***	2.202 ***	2.316 ***	2.140 ***
	(0.572)	(0.529)	(0.481)	(0.472)	(0.434)	(0.481)	(0.517)	(0.443)
Income elasticities for various foods								
F1	−0.528 ***	−0.555 ***	−0.664 ***	−0.737 ***	−0.827 ***	−1.125 ***	−0.633 ***	−0.840 ***
	(0.148)	(0.146)	(0.139)	(0.146)	(0.153)	(0.192)	(0.147)	(0.154)
F2	0.772 ***	0.711 ***	0.614 ***	0.507 ***	0.382 ***	0.409 ***	0.672 ***	0.430 ***
	(0.148)	(0.146)	(0.132)	(0.097)	(0.072)	(0.082)	(0.130)	(0.081)
F3	1.223 ***	1.054 ***	0.860 ***	0.757 ***	0.560 ***	0.561 ***	1.022 ***	0.637 ***
	(0.277)	(0.222)	(0.163)	(0.159)	(0.111)	(0.103)	(0.219)	(0.130)
F4	3.312 ***	2.810 ***	2.005 ***	1.357 ***	0.939 ***	0.948 ***	2.499 ***	1.071 ***
	(0.798)	(0.650)	(0.444)	(0.274)	(0.172)	(0.180)	(0.578)	(0.202)
F5	1.259 ***	1.106 ***	0.914 ***	0.767 ***	0.564 ***	0.594 ***	1.032 ***	0.635 ***
	(0.334)	(0.280)	(0.216)	(0.185)	(0.127)	(0.137)	(0.253)	(0.145)
F6	1.695 ***	1.478 ***	1.219 ***	0.995 ***	0.732 ***	0.806 ***	1.397 ***	0.828 ***
	(0.397)	(0.335)	(0.262)	(0.215)	(0.149)	(0.176)	(0.312)	(0.171)

Note: Standard errors are given in parentheses. *** *p* < 0.01. F1 = staple foods; F2 = vegetables and fruits; F3 = pork; F4 = beef and mutton; F5 = poultry and eggs; F6 = aquatic products.

**Table 4:** Own-price elasticities for various food groups from 2000 to 2020.

Year	2000	2004	2008	2012	2016	2020	2000–2010	2010–2020
Uncompensated own-price elasticities								
F1	−0.636 ***	−0.607 ***	−0.071	0.185	0.230	−0.336	−0.387	0.134
	(0.189)	(0.178)	(0.297)	(0.440)	(0.520)	(0.690)	(0.236)	(0.479)
F2	−0.584 ***	−0.547 ***	−0.521 ***	−0.583 ***	−0.595 ***	−0.571 ***	−0.577 ***	−0.594 ***
	(0.121)	(0.124)	(0.127)	(0.115)	(0.120)	(0.131)	(0.119)	(0.120)
F3	−0.666 ***	−0.719 ***	−0.731 ***	−0.657 ***	−0.694 ***	−0.795 ***	−0.679 ***	−0.683 ***
	(0.115)	(0.103)	(0.066)	(0.062)	(0.070)	(0.090)	(0.089)	(0.072)
F4	−0.947 **	−0.946 **	−0.806 ***	−0.837 ***	−0.901 ***	−1.005 ***	−0.864 ***	−0.901 ***
	(0.305)	(0.290)	(0.176)	(0.133)	(0.109)	(0.123)	(0.224)	(0.112)
F5	−0.043	−0.125	−0.188	−0.163	−0.228	−0.270	−0.156	−0.227
	(0.241)	(0.218)	(0.186)	(0.177)	(0.179)	(0.182)	(0.199)	(0.174)
F6	−1.513 ***	−1.485 ***	−1.389 ***	−1.370 ***	−1.360 ***	−1.399 ***	−1.444 ***	−1.367 ***
	(0.350)	(0.320)	(0.292)	(0.284)	(0.268)	(0.291)	(0.311)	(0.271)
Compensated own-price elasticities								
F1	−0.840 ***	−0.827 ***	−0.330	−0.106	−0.103	−0.691	−0.629 **	−0.189
	(0.170)	(0.169)	(0.269)	(0.404)	(0.481)	(0.668)	(0.209)	(0.441)
F2	−0.176 **	−0.166 **	−0.158 *	−0.176 **	−0.179 **	−0.178 **	−0.174 **	−0.179 **
	(0.058)	(0.062)	(0.066)	(0.055)	(0.056)	(0.067)	(0.058)	(0.056)
F3	−0.336 ***	−0.362 ***	−0.342 ***	−0.299 ***	−0.324 ***	−0.384 ***	−0.331 ***	−0.319 ***
	(0.057)	(0.049)	(0.046)	(0.062)	(0.047)	(0.036)	(0.048)	(0.049)
F4	−0.780 **	−0.775 **	−0.625 ***	−0.638 ***	−0.692 ***	−0.790 ***	−0.690 **	−0.694 ***
	(0.291)	(0.274)	(0.182)	(0.147)	(0.119)	(0.117)	(0.219)	(0.121)
F5	0.108	0.033	−0.021	0.003	−0.055	−0.093	0.007	−0.054
	(0.219)	(0.197)	(0.167)	(0.164)	(0.161)	(0.163)	(0.180)	(0.157)
F6	−1.364 ***	−1.332 ***	−1.230 ***	−1.208 ***	−1.195 ***	−1.239 ***	−1.290 ***	−1.203 ***
	(0.346)	(0.315)	(0.293)	(0.285)	(0.268)	(0.288)	(0.309)	(0.272)

Note: Standard errors are given in parentheses. * *p* < 0.10; ** *p* < 0.05; *** *p* < 0.01. F1 = staple foods; F2 = vegetables and fruits; F3 = pork; F4 = beef and mutton; F5 = poultry and eggs; F6 = aquatic products.

**Table 5:** Three scenarios regarding income elasticities of individual food groups.

Food Groups	Scenarios		
	S1	S2	S3
Staple foods	−0.772	−0.840	−0.924
Vegetables and fruits	0.578	0.430	0.387
Pork	0.867	0.637	0.574
Beef and mutton	1.696	1.071	0.964
Poultry and eggs	0.870	0.635	0.571
Aquatic products	1.155	0.828	0.745

**Table 6:** The projected impacts of income growth on food demands and GHGEs (kg).

Food Groups	F (2020)	GHGEs (2020)	S1		S2		S3	
			ΔF	ΔGHGEs	ΔF	ΔGHGEs	ΔF	ΔGHGEs
Staple foods	141.2	172.0	−1.090	−1.328	−1.186	−1.444	−1.304	−1.589
Vegetables and fruits	155.0	69.6	0.896	0.402	0.666	0.299	0.600	0.269
Pork	18.2	52.6	0.158	0.456	0.116	0.335	0.104	0.302
Beef and mutton	3.5	74.4	0.059	1.262	0.038	0.797	0.034	0.718
Poultry and eggs	25.5	29.3	0.222	0.255	0.162	0.186	0.146	0.168
Aquatic products	13.9	7.5	0.161	0.087	0.115	0.062	0.104	0.056
Total		405.4		1.135		0.236		−0.076

Note: F represents food consumption; ΔF and ΔGHGEs represent the change in food consumption and corresponding agricultural GHGEs, respectively.

**Table 7:** The projected impacts of a rise in animal-based food prices on food demands and GHGEs (kg).

Food Groups	SS1		SS2		SS3		SS4	
	ΔF	ΔGHGEs	ΔF	ΔGHGEs	ΔF	ΔGHGEs	ΔF	ΔGHGEs
Staple foods	0.474	0.578	−0.056	−0.069	0.106	0.129	1.149	1.400
Vegetables and fruits	−0.276	−0.124	−0.051	−0.023	−0.350	−0.157	−0.236	−0.106
Pork	−0.124	−0.359	0.003	0.008	0.004	0.012	−0.042	−0.120
Beef and mutton	−0.017	−0.354	−0.031	−0.664	−0.011	−0.228	0.018	0.380
Poultry and eggs	−0.039	−0.045	0.000	0.000	−0.049	−0.057	−0.129	−0.148
Aquatic products	−0.045	−0.024	0.020	0.011	0.001	0.001	−0.195	−0.105
Total		−0.328		−0.737		−0.301		1.300

Note: F represents food consumption; ΔF and ΔGHGEs represent the change in food consumption and related agricultural GHGEs, respectively. SS1 increases pork prices by 1%; SS2 increases beef and mutton prices by 1%; SS3 increases poultry and egg prices by 1%; SS4 increases aquatic products prices by 1%.

## Data Availability

The data on food consumption, demographic, and economic information can be found at http://www.stats.gov.cn/tjsj./ndsj/ (accessed on 1 June 2022). The data on food prices are not public.

## Appendix A

**Table A1:** Average Uncompensated Elasticities for Various Foods between 2000–2020.

Food Groups	F1	F2	F3	F4	F5	F6
F1	−0.200	0.330	0.336 **	−0.040	0.075	0.814 *
	(0.322)	(0.208)	(0.106)	(0.115)	(0.201)	(0.336)
F2	−0.181	−0.585 ***	−0.178 ***	−0.033	−0.226 **	−0.152
	(0.103)	(0.120)	(0.043)	(0.043)	(0.082)	(0.126)
F3	−0.019	−0.579 **	−0.683 ***	0.015	0.022	−0.229
	(0.195)	(0.214)	(0.081)	(0.089)	(0.171)	(0.264)
F4	−0.685	−0.568	−0.476 **	−0.892 ***	−0.307	0.510
	(0.412)	(0.380)	(0.153)	(0.151)	(0.274)	(0.444)
F5	−0.404	−0.147	−0.152	0.001	−0.194	−0.505
	(0.231)	(0.221)	(0.078)	(0.097)	(0.186)	(0.287)
F6	−0.013	−0.606 *	−0.321 **	0.141	0.010	−1.405 ***
	(0.252)	(0.282)	(0.110)	(0.100)	(0.188)	(0.290)

Note: Standard errors are given in parentheses. * *p* < 0.10; ** *p* < 0.05; *** *p* < 0.01. F1 = staple foods; F2 = vegetables and fruits; F3 = pork; F4 = beef and mutton; F5 = poultry and eggs; F6 = aquatic products.

## Appendix B

**Table A2:** The Change Trends of Income and Own-Price Elasticities in Previous Studies.

References	Food Groups	Year					Methods	Level
		1986	1990	2000	2010	2020		
Income elasticities								
[38]	Rice	0.290	0.270	0.180			LA/AIDS	Urban (household data)
	Flour	0.240	0.090	0.210				
	Pork	0.420	0.390	0.220				
	Beef and mutton	0.450	0.490	0.270				
	Poultry	0.480	0.500	0.260				
[26]	General cereals			0.403	0.266	0.174	Meta-regression	National (literature analysis)
	Pork			0.499	0.383	0.312		
	Poultry			0.501	0.409	0.348		
	Beef and mutton			0.514	0.421	0.359		
[40]	Grains			0.441	0.285		QUAIDS	Urban (household data)
	Livestock			0.536	0.414			
	Poultry			0.657	0.425			
	Eggs			0.581	0.448			
	Aquatic products			0.695	0.782			
	Vegetables			0.377	0.401			
	Fruits			0.603	0.434			
Own-price elasticities								
[38]	Rice	−0.300	−0.070	−0.680			LA/AIDS	Urban (household data)
	Flour	−0.810	−0.820	−1.230				
	Pork	−0.730	−0.620	−0.500				
	Beef and mutton	−1.080	−1.020	−0.690				
	Poultry	−0.790	−0.830	−0.700				

Note: QUAIDS = Quadratic Almost Ideal Demand System Model.
